# Myoepithelial Carcinoma Mimicking Basal Cell Carcinoma: A Case Report

**DOI:** 10.7759/cureus.95526

**Published:** 2025-10-27

**Authors:** Farlin Asharaff, Neena Nayak, Roger Webb, Karwan Moutasim, Soogan Lalla

**Affiliations:** 1 Dermatology, University Hospital Southampton NHS Foundation Trust, Southampton, GBR; 2 Maxillofacial Surgery, University Hospital Southampton NHS Foundation Trust, Southampton, GBR; 3 Histopathology, University Hospital Southampton NHS Foundation Trust, Southampton, GBR

**Keywords:** basal cell neoplasms, cancer, dermatopathology, epithelial-myoepithelial carcinoma, maxillofacial surgery, sweat gland

## Abstract

We present the case of an 81-year-old male with a two-month history of a rapidly growing red nodule measuring 6 mm x 10 mm on his left malar cheek. Histological examination confirmed the diagnosis of myoepithelial carcinoma. The nodule was fully excised with a 3.8 mm radial margin and a close deep margin of 0.95 mm. This was followed by a wide local excision with a 5 mm margin, including subcutaneous fat up to the muscles of facial expression, and closed with a lateral cheek advancement flap per the specialist skin multidisciplinary team (SSMDT) recommendation. Postoperatively, the patient has been followed up as per the high-risk squamous cell carcinoma protocol, including four-monthly clinic reviews in the first year and six-monthly reviews in the second year. This case highlights the importance of raising awareness around cutaneous myoepithelial carcinoma's rare presentation and diagnostic challenges, and the importance of multidisciplinary management with long-term follow-up.

## Introduction

Cutaneous myoepithelial carcinoma (CMC), also known as malignant myoepithelioma, is a rare form of cutaneous malignancy. It arises from contractile myoepithelial cells located between the basement membrane and luminal cells of glandular or secretory structures such as sweat glands [1]. These cells exhibit bidirectional differentiation, expressing both epithelial and mesenchymal (myoid) markers, reflecting their complex biological origin and behavior. They also play a key role in sweat transport [2,3]. Myoepithelial tumors frequently develop from pre-existing pleomorphic adenomas following malignant transformation, particularly in salivary glands and soft tissues, with primary cutaneous manifestations being exceedingly rare [4].

Cutaneous myoepithelial carcinoma usually presents as a solitary, slowly enlarging dermal or subcutaneous nodule, most frequently arising on the extremities or within the head and neck region. Additional sites reported in the literature include the axilla, back, thigh, hip, and feet [5]. It clinically resembles more common skin neoplasms, including squamous cell carcinoma, adnexal tumors, benign mixed tumors, or fibrous papules [6]. Given its rarity and varied presentation, it is often subject to diagnostic delays or misdiagnosis.

Despite its often-indolent clinical appearance, CMC can behave aggressively, with a notable risk of local recurrence, deep tissue invasion, and, in some cases, distant metastasis [7]. Due to this aggressive behavior, optimal management often necessitates a multimodal approach, including surgical excision, chemotherapy, radiotherapy, and, in select cases, systemic treatment to improve clinical and prognostic outcomes [8].

Myoepithelial carcinoma (MEC) has been reported to exhibit a slight female predominance and a bimodal age distribution, most commonly affecting children or adults between the fifth and sixth decades of life [9]. On the other hand, basal cell carcinoma (BCC) is the most common cutaneous malignancy, arising from basal keratinocytes and strongly linked to chronic ultraviolet (UV) exposure. It typically presents as a slow-growing, pearly papule on sun-exposed areas, especially the face and neck. While locally invasive, BCC rarely metastasizes and has an excellent prognosis with early treatment [10].

Here, we present a rare case of CMC on the left malar cheek in an 81-year-old male, clinically mimicking BCC at initial assessment. This case report aims to contribute to the literature by illustrating the importance of timely diagnosis and regular surveillance.

## Case presentation

**Figure 1:**
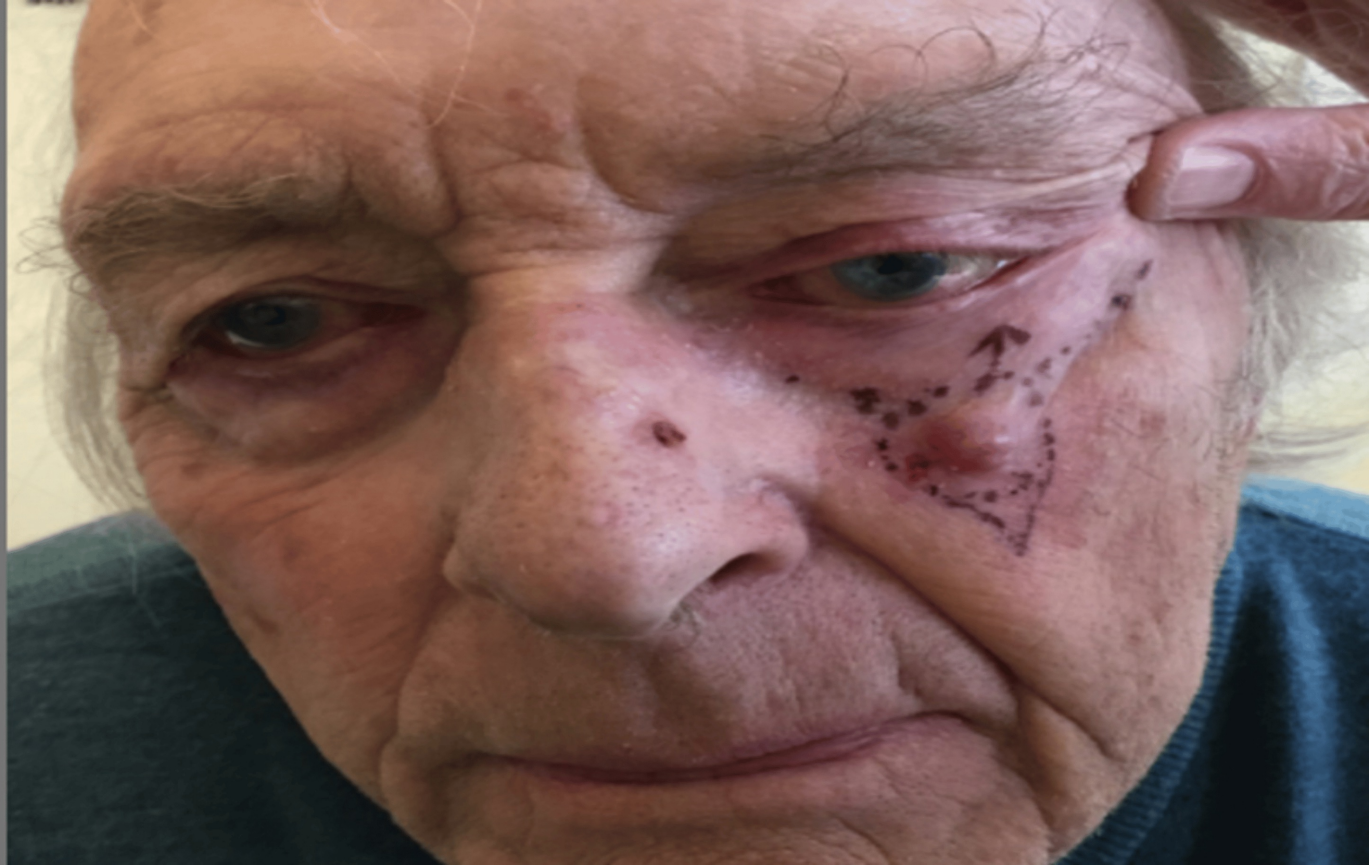
Preoperative clinical picture of the lesion on the left malar cheek

An 81-year-old male presented to the urgent skin cancer dermatology clinic with a two-month history of a rapidly growing red nodule 6 mm x 10 mm on his left malar cheek (Figure 1). There was no regional lymphadenopathy. His medical history included BCC on his left cheek, Bowen’s disease on his left cheek and temple, a previous deep vein thrombosis, pulmonary embolism, hypertension, cataracts, and a permanent pacemaker secondary to heart block. His regular medications were rivaroxaban, indapamide, mirtazapine, and ramipril. The nodule was clinically diagnosed as a BCC and subsequently excised under a local anesthetic. The tumor was excised down to the fascial superficial musculoaponeurotic system (SMAS) layer, and the defect was closed with a small advancement flap.

Investigation

**Figure 2:**
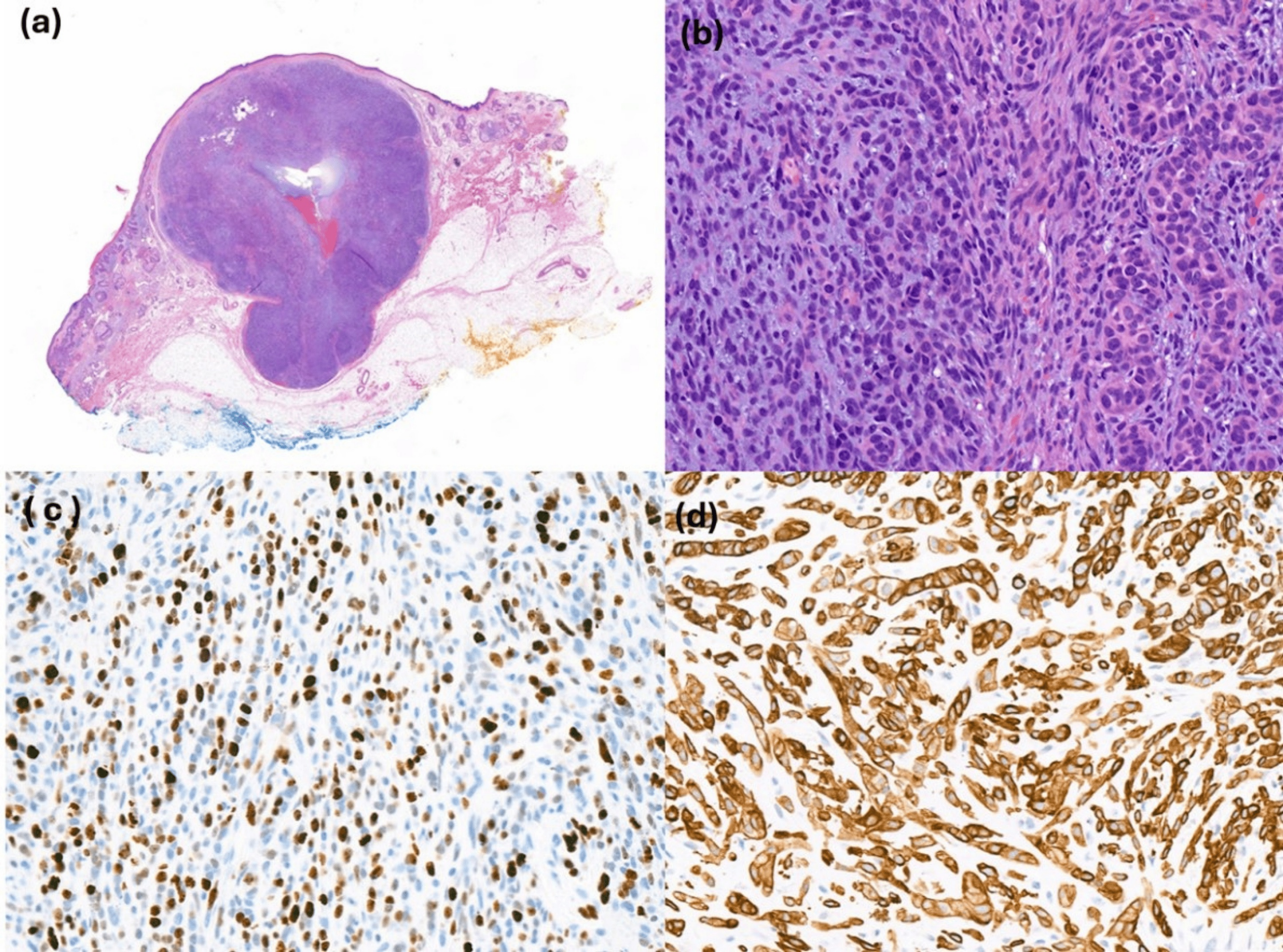
Histology pictures of the excision (a) Low power image (x10); (b) High power image (x40); (c) High expression of Ki67; (d) Positive staining for CK8/18

Histology confirmed an MEC. The excised specimen was a dermal-based tumor with a relatively circumscribed outline, measuring 11 mm in diameter, and comprised cellular sheets of spindled cells with admixed areas showing ductular differentiation focally. There was a mucoid stromal background. There was mitotic activity and moderate nuclear pleomorphism. No obvious squamous differentiation was seen. The tumor was 9 mm in thickness, filling the dermis and extending into the subcutis. There was no evidence of perineural or lymphovascular invasion (Figure 2, panels a and b).

On immunohistochemistry, the neoplastic cells were largely positive with S100 and Sox10 (myoepithelial markers), with negative Melan-A. There was scattered staining by the pan-cytokeratin cocktail AE1/AE3, whilst CK7 and CK20 appeared negative. Neuroendocrine markers were negative, and the MIB1 proliferation index was brisk (40% to 50%). There was high expression of Ki-67 (Figure 2, panel c). The CK8/18 showed variable but intense expression in the tumor cells (Figure 2, panel d). Patchy CK5/6 and MNF116 staining was also noted. There was nuclear preferentially expressed antigen in melanoma (PRAME) staining. Based on these findings, a diagnosis of MEC was made. Molecular analysis did not detect a clinically actionable rearrangement of the BRAF, EWSR1, and PLAG genes. The percentage of neoplastic cells was estimated to be 50% in the formalin-fixed paraffin-embedded (FFPE) sections provided.

Treatment and follow-up

The nodule was fully excised with a 3.8 mm radial margin and a close deep margin of 0.95 mm. A wide local excision was recommended by the specialist skin multidisciplinary team (SSMDT) meeting to prevent recurrence. This was undertaken by the maxillofacial surgery team with a 5 mm margin, including subcutaneous fat up to the muscles of facial expression, and closed with a lateral cheek advancement flap. Final histopathology of this lesion confirmed complete excision of the tumor. Per the recommendation of the SSMDT, the patient has been followed up according to the high-risk squamous cell carcinoma protocol, including four-monthly clinic reviews in the first year and six-monthly in the second year. His annual CT chest, abdomen, and pelvis with contrast has been unremarkable so far.

## Discussion

In CMC, tumor cells often exhibit a broad spectrum of morphologies, including spindle-shaped, hyaline, clear cell, plasmacytoid, and epithelioid forms. Due to these variations in cell morphology and similarity in histologic appearance and immunohistochemical staining to their benign counterpart, it is often challenging to diagnose CMC histologically [11]. A higher cellularity, nuclear pleomorphism, prominent nucleoli, presence of tumor necrosis, increased mitotic activity, and perineural and lymphovascular infiltration distinguish MEC from its benign counterpart, myoepithelioma [12]. Furthermore, Kutzner et al. proposed that benign myoepithelial tumors are well-circumscribed dermal nodules lacking epidermal connection, with solid growth, uniform ovoid cells, and pale eosinophilic cytoplasm within a hyalinized or myxoid stroma [13]. These histopathological criteria help with the diagnostic process.

In our case, malignant mixed tumor and malignant chondroid syringoma were among the histopathological differential diagnoses considered. Myoepitheliomas are part of a morphological continuum that spans from pure myoepithelioma to chondroid syringoma (also known as cutaneous mixed tumor) and parachordoma [14]. Mentzel et al. similarly report that cutaneous myoepithelial neoplasms constitute a histopathological spectrum, ranging from benign mixed tumors to myoepitheliomas and ultimately to malignant MEC [15].

Immunohistochemical analysis plays a crucial role in confirming the myoepithelial origin of the tumor. Positive immunoreactivity for myogenic markers such as calponin, smooth muscle actin (SMA), muscle-specific actin (MSA), and occasionally desmin, together with epithelial markers including epithelial membrane antigen (EMA), cytokeratins (CK5/6, CK7, and CK8/18), and pan-cytokeratin (KL-1/AE1/AE3), supports the diagnosis of MEC. Additional immunoreactivity for S-100 protein, glial fibrillary acidic protein (GFAP), vimentin, and calcitonin further reinforces the myoepithelial nature of the neoplasm [16,17].

Currently, there is no consensus on the optimal management of CMC due to its rarity. Wide local excision with free margins is generally considered the mainstay of treatment of these rare cutaneous malignancies. Mohs micrographic surgery, adjuvant radiotherapy, chemotherapy with carboplatin and paclitaxel, and sentinel lymph node resection are other treatment options that have been reported [5]. There is also a report of a patient being enrolled in phase one of the clinical trial of a checkpoint kinase 1 inhibitor [17]. Furthermore, Guidry et al. suggest genetic testing to identify potentially effective systemic therapies, as they report a patient with a BRAF V600E mutation who responded to combined BRAF/MEK inhibitor therapy [18]. Unfortunately, appropriate initial treatment is not always performed in a timely manner owing to disease rarity and the difficulty of making a diagnosis. To our knowledge, not more than 16 cases of primary CMC have been reported in the literature [9].

## Conclusions

This case report aims to raise awareness of CMC and highlights the critical importance of maintaining a high index of suspicion, especially in lesions demonstrating rapid growth or unusual histopathological features. Comprehensive immunohistochemical profiling is essential for accurate diagnosis, distinguishing MEC from its benign counterparts and other cutaneous malignancies. Optimal management relies on complete surgical excision with clear margins, often necessitating multidisciplinary collaboration to tailor treatment and reconstructive strategies. Given the tumor’s potential for local recurrence and aggressive behavior, vigilant long-term follow-up is warranted, including regular clinical reviews and imaging as indicated. Furthermore, the rarity of CMC underscores the need for increased clinical awareness and further accumulation of case reports to better understand its biological behavior and inform evidence-based management protocols. This report also contributes valuable clinical and pathological insights that may assist clinicians and pathologists in early recognition and effective treatment of this uncommon cutaneous malignancy and underscores the need for pathologists to consider MEC in the differential diagnosis of cutaneous neoplasms exhibiting both epithelioid and spindle cell features.
